# Sevoflurane exposure may cause dysplasia of dendritic spines and result in fine motor dysfunction in developing mouse through the PI3K/AKT/mTOR pathway

**DOI:** 10.3389/fnins.2022.1006175

**Published:** 2022-09-28

**Authors:** Linhong Zhong, Xiaofan Ma, Yixuan Niu, Lei Zhang, Zhenyu Xue, Jia Yan, Hong Jiang

**Affiliations:** Department of Anesthesiology, Shanghai Ninth People’s Hospital, Shanghai Jiao Tong University School of Medicine, Shanghai, China

**Keywords:** sevoflurane, dendritic spine, PI3k/AKT/mTOR pathway, fine motor dysfunction, developing brain

## Abstract

Sevoflurane has become one of the most widely used volatile anesthetics in pediatric surgery. However, sevoflurane exposure may interfere with dendritic development and synaptogenesis, resulting in brain function impairment. The PI3K/AKT/mTOR pathway plays an important role in dendritic development and synaptic plasticity. Here we investigated whether sevoflurane exposure would affect the morphological proportions of dendritic spines in developing mouse and explored the role of the change of plasticity of dendritic spines in sevoflurane-induced neurodevelopmental toxicity. The related signaling pathway was also examined. C57BL/6 mice at postnatal day (PND) 7 were exposed to 2% sevoflurane for 3 h. The PI3k/AKT/mTOR agonist IGF-1 or the mTOR phosphorylation inhibitor KU0063794 was intraperitoneally injected 30 min before sevoflurane or O_2_ exposure at PND7. Hippocampi were harvested 6 h after sevoflurane exposure. Western blotting was applied to measure the protein expression of PI3K/AKT/mTOR pathway phosphorylation. At PND14, brains from all groups were harvested for Golgi staining, and the morphology of dendritic spines of hippocampal neurons was observed by an oil immersion lens. When the mice grew to adolescence (PND48), fine motor function was measured by the Beam walking test. Here we showed that exposure to 2% sevoflurane for 3 h decreased the proportion of thin dendritic spines and increased the proportion of mushroom dendritic spines, but not changed the density of the dendritic spines. Sevoflurane exposure also suppressed the phosphorylation of the PI3K/AKT/mTOR pathway in immature mice hippocampi, and eventually led to long-term fine motor dysfunction. Meanwhile, IGF-1 pretreatment could rescue and KU0063794 pretreatment could aggravate the impairment induced by sevoflurane. In conclusion, sevoflurane exposure may cause a change of proportions of the types of dendritic spines through impacting the phosphorylation expression of the PI3K/AKT/mTOR pathway, and eventually led to long-term fine motor dysfunction in developing mouse.

## Introduction

Every year, millions of infants and children receive surgery under anesthesia. The safety of brain development under anesthesia is directly related to the quality of the population and is one of the public health issues concerned by the whole society (Psaty et al., 2015). A recent U.S. Food and Drug Administration (FDA) warning advised that exposure to general anesthetics may affect neurodevelopment in children (Davidson and Sun, 2018). Unfortunately, to date, no effective strategies exist to avoid to this problem.

Sevoflurane is one of the most widely used volatile anesthetics in pediatric anesthesia. In developing animals particularly in rodents and monkeys, receiving prolonged sevoflurane exposure induces neuronal apoptosis (He et al., 2018; Zhao et al., 2020; Zhu et al., 2021), abnormal development of the dendritic spine (Briner et al., 2010) and synapse formation and loss (Vutskits and Xie, 2016). Increasing evidence demonstrate that sevoflurane exposure induces neuronal apoptosis and subsequently leads to motor and cognitive dysfunction (Kalkman et al., 2009; Wilder et al., 2009; Sun, 2010; Flick et al., 2011; Sprung et al., 2012; Stratmann et al., 2014). Clinically, exposure to low concentration (2%) of sevoflurane for 3 h is closer to the practical approach to pediatric anesthesia (Zou et al., 2009). It was found that exposure to 2% sevoflurane for 3 h did not induce neuroapoptosis, but could cause long-term abnormal behavior (Lu et al., 2016). It suggests the mechanisms of immature brain injury induced by sevoflurane anesthesia are various.

Neurogenesis, especially dendritic spine morphogenesis, is a key step in brain circuits during the development of the central nervous system (Liao et al., 2021). During the peak period of brain development, the development of the dendritic spine also reaches its peak. In this period, the abnormal morphological plasticity of dendritic spines will trigger disturbances in synapse formation, which will lead to long-term neurodevelopmental dysfunction (Bian et al., 2015). Dendritic spines can be divided into four types: mushroom, thin, stubby and branched (Harris et al., 1992). Studies have confirmed that a change in the proportions of the types of dendritic spines would predict a decrease in learning ability (Bello-Medina et al., 2016). The morphological plasticity of dendritic spines is not only related to learning and memory function, but also closely related to motor coordination. Previous studies have found that neuroplasticity is the basis of the brain’s restorative response to environmental factors that impair neurobiological processes. Neuropathological lesions resulting in motor dysfunction are usually accompanied with neuroplasticity change (Gonzalez-Burgos et al., 2011).

The phosphatidylinositol 3-hydroxy kinase/protein kinase B/mammalian target of the rapamycin (PI3K/AKT/mTOR) pathway plays an important role in different neuronal functions (Gilbert and Man, 2017; Sharma and Mehan, 2021). Several studies have shown that the phosphorylation expression of the PI3K/AKT/mTOR pathway involved in different neuronal functions mainly involves synaptogenesis, corticogenesis, and related neuronal cerebral processes, especially in the hippocampus (Sharma and Mehan, 2021). As the key component of the pathways, the importance of mTOR is self-evident. PI3K/AKT, the upstream signal of mTOR, plays an important regulatory role in the activation of mTOR (Kim et al., 2017).

In this study, we investigated whether exposure to 2% sevoflurane for 3 h would affect the morphological plasticity of dendritic spines in developing mouse, and explored whether PI3K/AKT/mTOR pathway played a key role in sevoflurane-induced immature neurotoxicity.

## Materials and methods

### Animals and animal treatments

This study was approved by the Animal Ethics Committee of Shanghai Ninth People’s Hospital, Shanghai Jiao Tong University School of Medicine and was performed in compliance with the Guide for the Care and Use of Laboratory Animals of the institution. C57BL/6 mouse pups with the age of postnatal day 7 (PND7), provided by the Animal Center of Shanghai Jiao Tong University School of Medicine (Shanghai, China), were used. The animals were housed on a 12 h light/dark cycle and with free access to food and water.

Mouse pups were separated from their mothers for acclimatization before sevoflurane exposure. Mouse pups were exposed to 2% sevoflurane for 3 h or 100% oxygen for 3 h in a chamber. The concentration of sevoflurane in the chamber was monitored and maintained by a vaporizer. The gas flow to the chamber was 2 L/min. Other studies have shown that PND7 C57BL/6 mice can be exposed to up to 3% sevoflurane for 6 h without any detrimental effect on cerebral perfusion, arterial blood gas, or glucose values (Satomoto et al., 2009). After exposure, the mouse pups were returned to the cages of mothers for further experiments.

**FIGURE 1:**
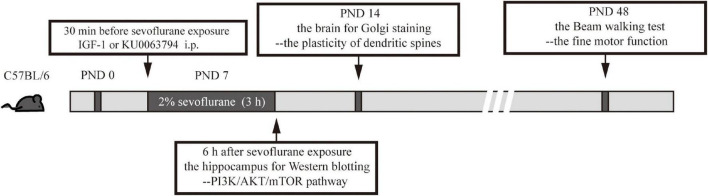
The overall experimental procedures in this study.

In the brain, the time to reach the effective concentration of IGF-1 (a phosphorylated activator for PI3K/AKT/mTOR) was from 30 min to 2 h (Guan et al., 2015) and that of KU0063794 (a second generation mTOR inhibitor targeting mTORC1 and mTORC2) was from 10 min to 4 h after intraperitoneal (i.p.) injection (Garcia-Martinez et al., 2009). Therefore, to ensure the effectiveness of these drugs during anesthesia, the mice received IGF-1 or KU0063794 i.p. 30 min before exposure to sevoflurane or O_2_. IGF-1 was dissolved in saline, which possesses the ability to cross the blood brain barrier (BBB) according to the previous report (Nishijima et al., 2010). KU0063794 was dissolved in DMSO. We used 240 μg/kg IGF-1 (Yuan et al., 2020) or 5 mg/kg KU-0063794 (Cordaro et al., 2017; Campolo et al., 2021) as previously reported. As the solute group, the mice received the equivalent amount of saline or DMSO with i.p. injection 30 min before exposure to sevoflurane or O_2_. The overall experimental procedures were shown in Figure 1.

### Western blotting

The mouse hippocampus was isolated immediately on ice after being sacrificed by decapitation 6 h after exposure to sevoflurane for western blotting. In brief, tissues were lysed and homogenized in a buffer containing a protease inhibitor and phosphatase inhibitor cocktail (Epizyme, Shanghai, China). The homogenate was centrifuged and the supernatant was collected for further analysis. The protein concentrations of samples were determined using the BCA protein assay (Beyotime, Shanghai, China). Equal amounts of protein were boiled in loading buffer (Sangon Biotech, Shanghai, China) and separated by 8% polyacrylamide gel electrophoresis, and transferred onto PVDF membranes (Millipore, Burlington, MA, USA). The membranes were incubated at room temperature for 2 h in a blocking buffer. Protein sample were subjected to western blot analysis using the following primary antibodies: anti-mTOR (1:10,000, Abcam), anti-phospho-mTOR (1:1,000, Abcam), anti-PI3K (1:1,000, Abcam), anti- phospho-PI3K (1:1,000, Abcam), anti-AKT (1:500, Abcam), anti-phospho-AKT (1:1,000, Abcam), and anti-β-actin (1:1,000, Cell Signaling Technology). The secondary antibodies were goat-anti-rabbit IgG or mouse-anti-goat IgG (1:3,000, Cell Signaling Technology). All blots were detected using the enhanced chemiluminescence (ECL) detection system (Millipore, MA, USA). The scanned images were quantified with Image J. We used β-actin concentrations to standardize amounts of protein.

### Golgi staining and dendritic spines counting

To observe the morphology of the dendritic spines, Golgi staining was performed after sevoflurane exposure. The brains were harvested at the age of postnatal day 14 (PND14). At PND14, dendritic spines have reached the peak development in mice (Fiala et al., 1998). Golgi staining was performed on 120 μm thick cryosectioned brain slices, using the FD Rapid Golgi Stain kit (FD NeuroTechnologies, Inc., Columbia, CA, USA) according to the manufacturer’s protocol (Du, 2019). For each hippocampal region, ten pyramidal neurons that were well-impregnated and separated from others were scanned with a confocal microscope (Nikon A1). To analysis the morphology of dendritic spines, five segments of 50 μm (or longer) dendrites were randomly chosen from each neuron with clearly trace of origin and well separated from neighboring dendrites and imaged with 100× oil immersion lens. Spine density was calculated as the number of spines per 10 μm of dendritic length.

The data on the morphology of the dendritic spine were analyzed by using the NeuroStudio^®^ Software (version 0.9.92; Computational Neurobiology and Imaging Center Mount Sinai School of Medicine, New York, NY, USA). The software can classify spines according to a default classification scheme. Morphological criteria based on the spine length (SL), neck diameter (ND), head diameter (HD), and the number of protrusions from a single stalk were used to classify the spines in thin (when HD/ND < 1.1 and SL/HD > 2.5), mushroom (when HD > 0.35 μm), branch (with a single stalk with two or more spine heads), and stubby (others) (Dall’oglio et al., 2015; Zancan et al., 2018). The data on the dendritic spine density were also collected. Dendritic spines density was calculated as the total number of spines divided by the length of the dendrite studied (Rasia-Filho et al., 2004). All the analysis was completed by two independent investigators who were blinded to the condition.

### Beam walking test

A protocol for behavioral assessment showed that mice could be tested for motor function, balance and coordination at 1 month of age (Rogers et al., 2001). Beam walking test was performed for testing fine motor coordination and balance of mice reaching PND 48 from all groups, as described before (Luong et al., 2011). Briefly, the beam apparatus consists of 1 m beams with a flat surface of 12 mm, resting 50 cm above the table top on two poles. A black box was placed at the end of the beam as the finish point. The time and performance to cross the center 80 cm were measured by a video camera. Mice were placed at one end of a beam and the time required to cross the escape box at the other end (80 cm away) was measured by a timer. The timer was started with the nose of the mice entering the center 80 cm, and stopped when the animal reached the end. This test takes place over three consecutive days. A slip was defined as the foot coming off the top of the beam. The times of their foot slipping off the beam were recorded. The beam was cleaned with 75% alcohol to avoid olfactory cues between two tests.

### Statistical analysis

All values are presented as means ± standard deviation. All the data were analyzed by one-way ANOVA analysis of variance followed by the Tukey’s test. The GraphPad Prism 9.0 (GraphPad, USA) software were used to conduct the statistical analysis. Statistical significance was accepted as *P* < 0.05.

## Results

### Exposure to sevoflurane in the neonatal period can alter the proportions of the types of dendritic spines, but not the density of the dendritic spines in young mice

**FIGURE 2:**
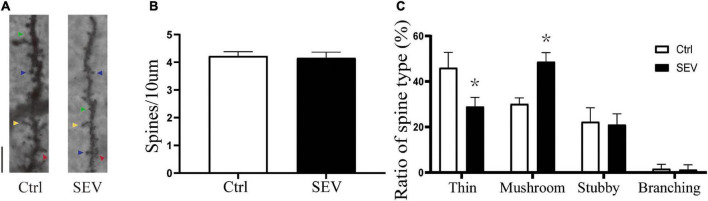
Sevoflurane can alert the proportions of the dendritic spines. **(A)** Representative images of Golgi staining of dendritic spines in the hippocampi of PND 14 mice. Different spine types are indicated by relevant color arrowheads: thin spine (yellow), mushroom spine (blue), stubby spine (green), branching spine (red). Scale bar = 10 μm. **(B,C)** The quantitative analysis of spine density and proportions of spine types by one-way ANOVA. Results are the means ± SD, *n* = 6 mice per group. *P < 0.05 vs. Ctrl.

To explore the possible effect of sevoflurane on the plasticity of dendritic spines, we first examined the morphology of dendritic spines in the hippocampus from PND14 after exposure to sevoflurane using Golgi staining (Figure 2A). There was no significant change in dendritic spine density (*P* > 0.05, Figure 2B). Notably, the proportion of mushroom dendritic spines increased in the sevoflurane (SEV) group (*P* < 0.05), and the proportion of thin dendritic spines decreased (*P* < 0.05), but the proportion of other types of dendritic spines had no significant change (*P* > 0.05), compared with the control (Ctrl) group (Figure 2C). These results suggested that sevoflurane could induce morphological change of dendritic spines in the hippocampus of young mice.

### Exposure to sevoflurane can suppress the phosphorylation of the PI3K/AKT/mTOR pathway in the hippocampus in neonatal mice

**FIGURE 3:**
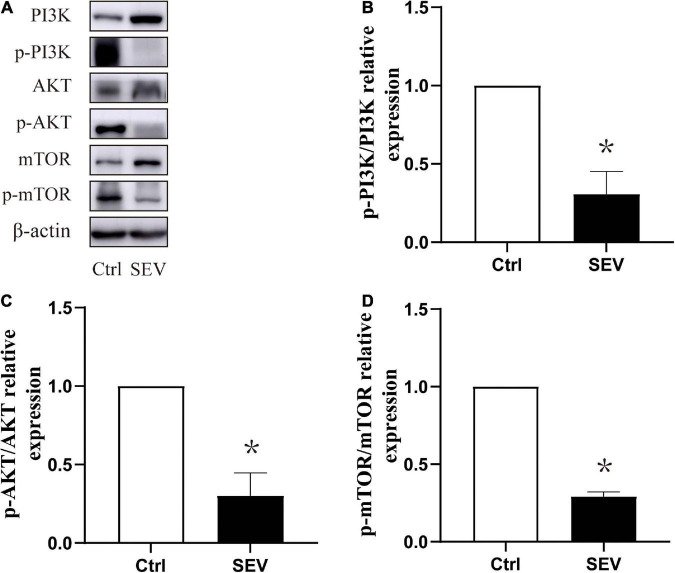
Sevoflurane can suppress the phosphorylation expression of the PI3K/AKT/mTOR pathway in the hippocampus in neonatal mice. **(A)** Representative Western blots of PI3K, AKT, and mTOR. **(B–D)** Bars represent normalization of target proteins were ratio of phosphorylated and non-phosphorylated forms. The quantitative analysis of PI3K, AKT and mTOR when exposure to sevoflurane after 6 h by one-way ANOVA. Results are the means ± SD. *n* = 6 in each group. *P < 0.05 vs. Ctrl.

To study whether the phosphorylation of the PI3K/AKT/mTOR pathway is involved in sevoflurane-induced morphological abnormality of dendritic spines, the expressions of the proteins were detected by western blot (Figure 3A). Compared with the Ctrl group, the phosphorylation of PI3K (*P* < 0.05, Figure 3B), AKT (*P* < 0.05, Figure 3C), and mTOR (*P* < 0.05, Figure 3D) were suppressed by the SEV group. These results indicated that exposure to 2% sevoflurane for 3 h could inhibit the phosphorylation of the PI3K/AKT/mTOR pathway.

### IGF-1 can alleviate the inhibition of PI3K/AKT/mTOR phosphorylation and rescue the morphological abnormality of dendritic spines induced by sevoflurane

**FIGURE 4:**
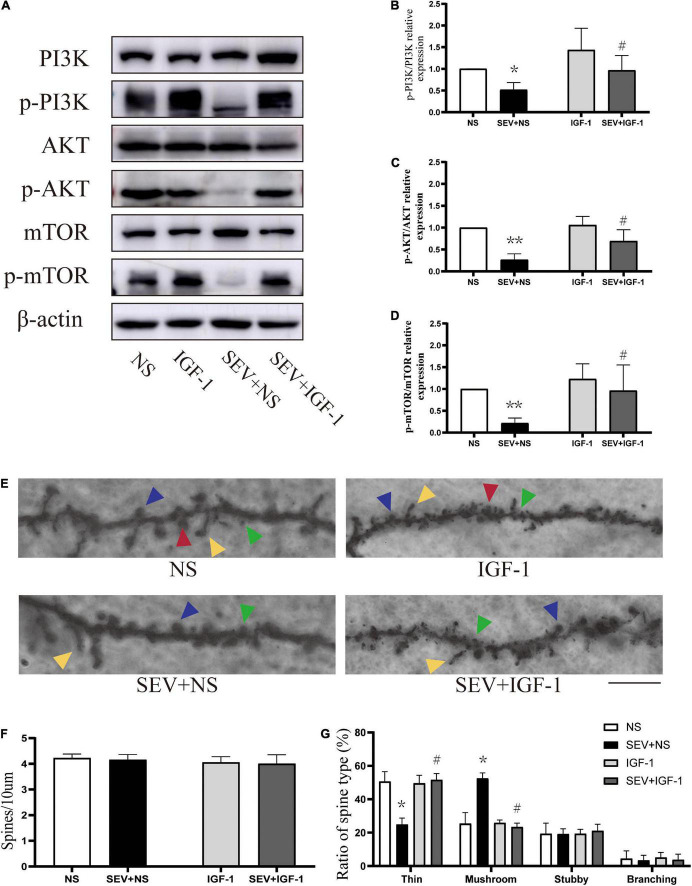
IGF-1 can alleviate the inhibition of PI3K/AKT/mTOR phosphorylation and rescue sevoflurane-induced morphological abnormality of dendritic spines. **(A)** Representative Western blot of PI3K, AKT, mTOR, and phosphor-PI3K, phosphor-AKT, phosphor-mTOR for different groups. β-actin was used as reference. **(B–D)** Bars represent normalization of target proteins were ratios of phosphorylated and non-phosphorylated forms. Results of one-way ANOVA and significance are shown inside graphs: data were presented as mean ± SD, *n* = 6 for per group. **(E)** Representative images of Golgi staining of dendritic spines in the hippocampi of PND 14 mice for different groups. Different spine types are indicated by relevant color arrowheads: thin spine (yellow), mushroom spine (blue), stubby spine (green), branching spine (red). Scale bar = 10 μm. **(F)** The density of the dendritic spines in different groups. **(G)** The proportions of dendritic spine types in different groups. Data were presented as mean ± SD by one-way ANOVA, *n* = 6 mice per group. *P < 0.05, **P < 0.01 vs. NS; ^#^*P* < 0.05 vs. SEV + NS.

To define the role of the pathway in sevoflurane-induced morphological change of dendritic spines in the hippocampus in young mice. We used the agonists of PI3K/AKT/mTOR pathway for stimulating signaling cascades. Compared with the control (NS) group, the phosphorylation of PI3K (*P* < 0.05, Figures 4A,B), AKT (*P* < 0.01, Figures 4A,C) and mTOR (*P* < 0.01, Figures 4A,D) was suppressed in the sevoflurane (SEV + NS) group. Treatment with IGF-1 before sevoflurane (SEV + IGF-1) group showed no significant difference in phosphorylation of PI3K/AKT/mTOR pathway, compared with the NS group (*P* > 0.05). However, the expression of phosphorylation of PI3K (*P* < 0.05, Figure 4B), AKT (*P* < 0.05, Figure 4C) and mTOR (*P* < 0.05, Figure 4D) was increased, compared with the SEV + NS group. It indicated that IGF-1 could rescue the inhibition of PI3K/AKT/mTOR phosphorylation pathway induced by sevoflurane.

In Golgi staining analysis (Figure 4E), there was no significant change in density of dendritic spines in each group (*P* > 0.05, Figure 4F). However, compared with the NS group, the proportion of mushroom dendritic spines increased (*P* < 0.05) and the proportion of thin dendritic spines decreased (*P* < 0.05), but the proportion of other types of dendritic spines had no significant change (*P* > 0.05) in the SEV + NS group (Figure 4G). Surprisingly, the decrease of the proportion of thin dendritic spines and increase of the proportion of mushroom dendritic spines were rescued by IGF-1 (*P* > 0.05, Figure 4G). These results suggested that sevoflurane-induced change of the proportions of the types of dendritic spines was mediated by the PI3K/AKT/mTOR phosphorylation pathway.

### KU0063794 can aggravate sevoflurane-induced morphological abnormality of dendritic spines

**FIGURE 5:**
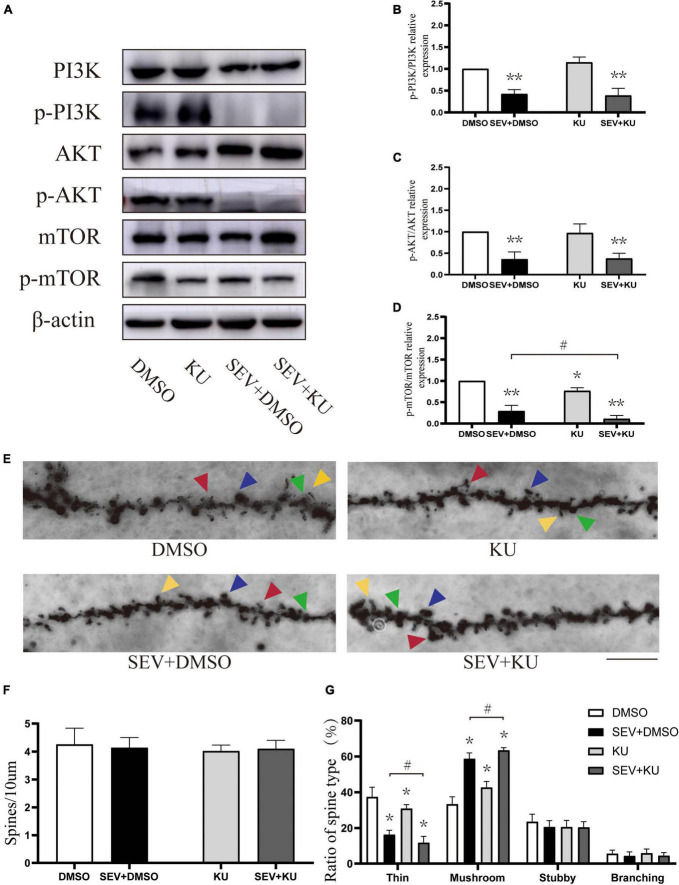
KU0063794 can aggravate sevoflurane-induced morphological abnormality of dendritic spines. **(A)** Representative Western blot of phosphorylated and non-phosphorylated proteins for different groups. β-actin was used as reference. **(B–D)** Normalization of phosphorylated and non-phosphorylated forms. Results of one-way ANOVA and significance are shown inside graphs: data were presented as mean ± SD, *n* = 6 for per group. **(E)** Representative images of Golgi staining of dendritic spines in the hippocampi of PND 14 mice in different groups. Different spine types are indicated by relevant color arrowheads: thin spine (yellow), mushroom spine (blue), stubby spine (green), branching spine (red). Scale bar = 10 μm. **(F)** The density of the dendritic spine in different groups. **(G)** The proportion of dendritic spine types in different groups. Data were presented as mean ± SD by one-way ANOVA, *n* = 6 mice per group. *P < 0.05, **P < 0.01 vs. DMSO; ^#^*P* < 0.05 vs. SEV + DMSO.

We used the inhibitor of mTOR to suppress the PI3K/AKT/mTOR signaling pathway (Figure 5A). Compared with the control (DMSO) group, the phosphorylation of PI3K (*P* < 0.01, Figure 5B), AKT (*P* < 0.01, Figure 5C), and mTOR (*P* < 0.01, Figure 5D) were suppressed in sevoflurane (SEV + DMSO) group. Moreover, the mTOR inhibitor administration before exposure to sevoflurane (SEV + KU group) could aggravate the inhibition of the phosphorylation of mTOR, compared with the SEV + DMSO group (*P* < 0.05, Figure 5D). Using the inhibitor of mTOR alone (KU group) significantly also reduced the phosphorylation of mTOR (*P* < 0.05, Figure 5D) and barely changed the phosphorylation of PI3K and AKT (*P* > 0.05, Figures 5B,C). In Golgi staining experiment (Figure 5E), although there was no significant change in density of dendritic spines in each group (*P* > 0.05, Figure 5F), the proportion of mushroom dendritic spines increased (*P* < 0.05) and the proportion of thin dendritic spines decreased (*P* < 0.05) in the SEV + DMSO group, compared with the DMSO group (Figure 5G). Compared with the SEV + DMSO group, the increase of proportion of mushroom dendritic spines (*P* < 0.05) and the decrease of proportion of thin dendritic spines were aggravated (*P* < 0.05) in the SEV + KU group, but the proportions of other types of dendritic spines had no significant change (*P* > 0.05) (Figure 5G). In addition, compared with the DMSO group, the proportion of mushroom dendritic spines increased (*P* < 0.05) and the proportion of thin dendritic spines (*P* < 0.05) deceased in the KU group (Figure 5G). Taken together, these results indicated that sevoflurane-induced morphological abnormality of dendritic spines was mediated by the inhibition of the phosphorylation of PI3K/AKT/mTOR pathway.

### Exposure to sevoflurane in neonatal period can induce long-term fine motor dysfunction which can be rescued by IGF-1 and KU0063794 can induce fine motor impairment which is similar as sevoflurane-induced

**FIGURE 6:**
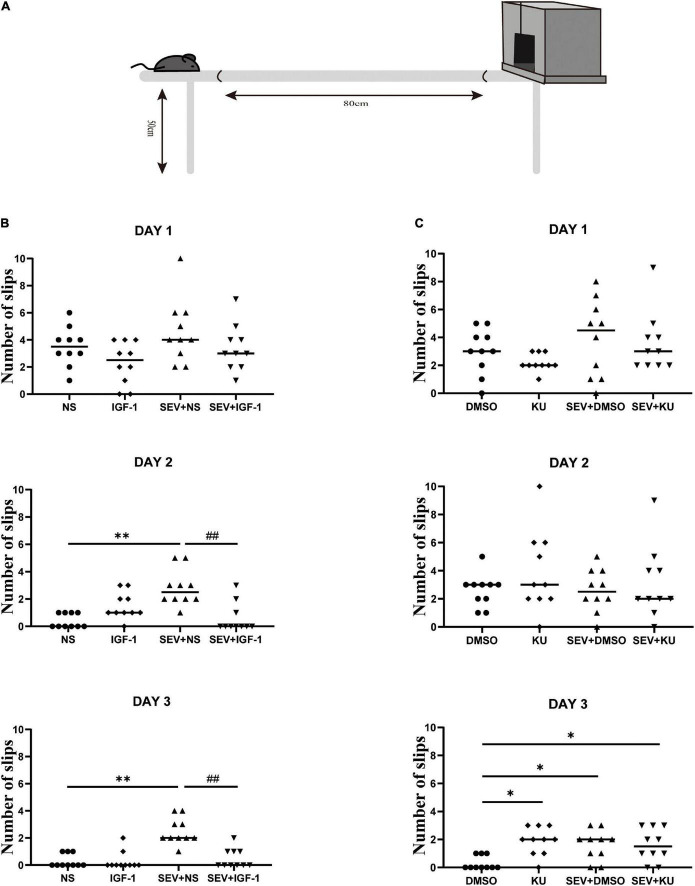
IGF-1 can rescue sevoflurane-induced deficits in fine motor function, and KU0063794 can induce fine motor impairment which is similar as sevoflurane-induced. **(A)** Schematic diagram of the beam walking test. **(B,C)** The numbers of slips of beam walking test at PND 48 over three consecutive days in all groups by one-way ANOVA, *n* = 10 for each group. *P < 0.05, **P < 0.01 vs. NS or DMSO; ^#^*P* < 0.05, ^##^*P* < 0.01 vs. SEV + NS.

Beam walking test is performed to detect fine motor coordination and balance function when the mice grew up (Figure 6A). Compared with the NS group, the number of slips significantly increased in the SEV + NS group (*P* < 0.01). The number of slips in the SEV + IGF-1 group was less than the SEV + NS group on the third day (*P* < 0.01, Figure 6B). Furthermore, compared with the DMSO group, both of the numbers of slips in the SEV + DMSO and SEV + KU group significantly increased on the third day (*P* < 0.05). Similar as SEV + DMSO group, the number of slips increased in the KU group at the third day (*P* < 0.05, Figure 6C). These results indicated that exposure to 2% sevoflurane for 3 h in the neonatal period could induce long-term fine motor dysfunction, which could be rescued by IGF-1. The inhibitor of mTOR also could induce similar impact as sevoflurane-induced.

## Discussion

Recent studies have found that a brief exposure to anesthesia in childhood do not damage the subsequent intellectual development (Ing et al., 2014; Sun et al., 2016). However, there are still evidence suggesting that exposure to anesthesia during infants and young children is associated with impaired motor skills and lower processing speed (Ing et al., 2014; Zaccariello et al., 2019). In the present study, we performed a series of experiments in a mouse model of exposure to 2% sevoflurane for 3 h to analyze the underlying mechanisms of sevoflurane-induced neurodevelopmental impairment. The result showed that exposure to 2% sevoflurane for 3 h could alter the proportions of the types of dendritic spines, but not the density of the dendritic spines. Also, sevoflurane exposure suppressed the phosphorylation expression of the PI3K/AKT/mTOR pathway in immature mouse hippocampus resulting in subsequent long-term fine motor dysfunction. IGF-1, which is the agonist of this pathway, can alleviate the inhibition of PI3K/AKT/mTOR phosphorylation and rescue the morphological abnormality of dendritic spines together with the fine motor dysfunction induced by sevoflurane. Moreover, the use of the inhibitor of mTOR phosphorylation aggravated the proportion alteration of the types of dendritic spines induced by sevoflurane, and finally led to long-term fine motor disorders as well.

Previous studies reported that in rodents’ early-life, long duration (6–9 h) exposure to high concentrations (3–4%) of sevoflurane could impair cognitive functions through apoptosis (Wang et al., 2013; Zhou et al., 2013). Meanwhile, it has also been shown in non-human primates that exposure to 2.5–3.5% sevoflurane for 5 h in neonatal rhesus monkey caused widespread apoptotic death of neurons and oligodendrocytes in the brain (Ikonomidou et al., 2019). Clinically, exposure to the lower concentration (2%) of sevoflurane for 3 h is closer to the practical approach to pediatric anesthesia (Zou et al., 2009). However, it was found that exposure to 2% sevoflurane for 3 h did not induce neuroapoptosis but could also cause long-term dysfunction in brain development (Lu et al., 2016). It suggests the mechanisms of immature brain injury induced by different concentrations and exposure durations of sevoflurane are not the same.

According to the function of spines, mushroom spines have larger spines, corresponding to stable and mature synapses, and are called “memory dendritic spines.” The thin spines, known as the “learning dendritic spines,” are more plastic and can be transformed into “memory dendritic spines” under the condition of long-term enhancement (LTP) caused by learning and other behaviors (Bourne and Harris, 2007). Dendritic spines show highly dynamic changes in morphology and number during development and adulthood (Yuste and Majewska, 2001). Under normal circumstances, the proportions of thin and mushroom spines keep a certain balance (Bourne and Harris, 2007). The decrease of “learning dendritic spines” and increase of “memory dendritic spines” predicted the impairment of neurodevelopment (Bello-Medina et al., 2016). Our data showed that although the total number of dendritic spines remained stable after exposure to sevoflurane, the proportions of thin and mushroom dendritic spines changed significantly, companying with fine motor dysfunction when the mice grew up. In the missing in metastasis (a protein initiates new dendritic spines) knockout mice, the decreased proportion of thin dendritic spines is associated with observed behavioral defects in motor-coordination (Minkeviciene et al., 2019). Therefore, we consider that sevoflurane exposure may alter the morphology of dendritic spines during neurodevelopment, and lead to long-term fine motor dysfunction.

The PI3K/AKT/mTOR pathway plays an important role in maintaining the plasticity of dendritic spines in the normal nervous system (Kumar et al., 2005). The general relevance of the PI3K/AKT/mTOR pathway in the regulation of dendritic growth and spine formation has been reported recently. It has been shown previously that the activation of the PI3K/AKT/mTOR pathway is required for the insulin action on the spine and synapse formation in hippocampal neurons (Lee et al., 2011). Our results suggested that sevoflurane-induced morphological abnormality of dendritic spines was dependent on the phosphorylation of the PI3k/AKT/mTOR pathway. Moreover, the results are consistent with the fact that mTOR is important in maintaining dendritic spine stability (Jaworski and Sheng, 2006; Tang et al., 2014; Chen et al., 2020). Although the density of dendritic spines is associated with dendritic growth and neurological diseases (Liu et al., 2019), our data showed that exposure to 2% sevoflurane for 3 h had no effect on the density of dendritic spines in the developing hippocampus which is consistent with Qiu et al. (2016).

Although the results suggested that the PI3K/AKT/mTOR pathway played an important role in the regulation of dendritic spines morphology in sevoflurane-induced neurotoxicity, how the PI3K/AKT/mTOR pathway trigger the morphological changes of dendritic spines remains unclear. In fact, Tau protein has been implicated as the PI3K/AKT/mTOR pathway downstream effector to control neurite outgrowth, and its hyperphosphorylation is considered to be the basis of neuronal degeneration in Alzheimer’s disease (Tao et al., 2014; Yang et al., 2020). A study has reported that Glutamate (Glu) can induce Tau phosphorylation in the postsynaptic structure by activating NMDA receptors under physiological conditions, thus promoting dendritic spines maturation (Pallas-Bazarra et al., 2016). Thus, we expect that further work will be performed to assess the role of Tau phosphorylation and NMDA receptor in the PI3K/AKT/mTOR pathway-mediating sevoflurane-induced dysplasia of dendritic spines.

In summary, our data showed that exposure to 2% sevoflurane for 3 h induces abnormal morphological proportions, but not density, of dendritic spines by impacting the phosphorylation of PI3K/AKT/mTOR pathway, which may be effect on developmental brain injury. It is expected to be of great significance and good application prospect in improving the safety of infant brain development during anesthesia.

## Data availability statement

The raw data supporting the conclusions of this article will be made available by the authors, without undue reservation.

## Ethics statement

The animal study was approved by the Animal Ethics Committee of Shanghai Ninth People’s Hospital, Shanghai Jiao Tong University School of Medicine (Shanghai, China) and was performed in compliance with the Guide for the Care and Use of Laboratory Animals of the institution.

## Author contributions

JY, HJ, and LZ: conceptualization. LZ, XM, YN, and ZX: methodology. LHZ: writing-original draft preparation. JY and HJ: writing-review and editing. All authors contributed to the article and approved the submitted version.
